# Chitosan Membrane Containing Copaiba Oil (*Copaifera* spp.) for Skin Wound Treatment

**DOI:** 10.3390/polym14010035

**Published:** 2021-12-23

**Authors:** Sheila Barbosa Paranhos, Elisângela da Silva Ferreira, Caio Augusto de Almeida Canelas, Simone Patrícia Aranha da Paz, Marcele Fonseca Passos, Carlos Emmerson Ferreira da Costa, Alisson Clay Rios da Silva, Sergio Neves Monteiro, Verônica Scarpini Candido

**Affiliations:** 1 Engineering of Natural Resources of the Amazon Program, Federal University of Pará—UFPA, Rua Augusto Correa 01, Belem 66075-110, Brazil; paranhos@ufpa.br (S.B.P.); licalipe8@yahoo.com.br (E.d.S.F.); paz@ufpa.br (S.P.A.d.P.); 2 Amazon Oil Laboratory, Faculty of Biotechnology, Federal University of Pará—UFPA, Rua Augusto Correa 01, Belem 66075-110, Brazil; caio.a.canelas@gmail.com; 3 Materials Science and Engineering Program, Federal University of Pará—UFPA, Tv We 26, Ananindeua 67130-660, Brazil; cellepassos@gmail.com (M.F.P.); alissonrios@ufpa.br (A.C.R.d.S.); 4 Chemical Program, Federal University of Pará—UFPA, Rua Augusto Correa 01, Belem 66075-110, Brazil; emmerson@ufpa.br; 5 Department of Materials Science, Military Institute of Engineering—IME, Praça General Tiburcio 80, Urca, Rio de Janeiro 22290-270, Brazil; snevesmonteiro@gmail.com

**Keywords:** polymers, chitosan, copaiba oil, wound healing

## Abstract

The interaction of copaiba oil in the polymer matrix of chitosan can produce a favorable synergistic effect and potentiate properties. Indeed, the bioactive principles present in copaiba oil have anti-inflammatory and healing action. In the present work, chitosan membranes containing different contents of copaiba oil copaíba (0.1, 0.5, 1.0 and 5.0% (*v*/*v*)) were for the first time investigated. The membranes were developed by the casting method and analyzed for their morphology, degree of intumescence, moisture content, contact angle, Scanning Electron Microscope, and X-ray diffractometry. These chitosan/copaiba oil porous membranes disclosed fluid absorption capacity, hydrophilic surface, and moisture. In addition, the results showed that chitosan membranes with the addition of 1.0% (v/*v*) of copaiba oil presented oil drops with larger diameters, around 123.78 μm. The highest fluid absorption indexes were observed in chitosan membranes containing 0.1 and 0.5% (*v*/*v*) of copaiba oil. In addition, the copaiba oil modified the crystalline structure of chitosan. Such characteristics are expected to favor wound treatment. However, biological studies are necessary for the safe use of chitosan/copaiba oil membrane as a biomaterial.

## 1. Introduction

Innovative dressings have been targeted by researchers to assist in the regeneration of damaged tissues. For efficient wound healing, it is necessary that the dressing is non-allergenic, non-toxic, maintains moisture, allows gas exchanges, protects the wound against pathogenic microorganisms, and absorbs exudates from the wound. Moreover, it must stimulate growth factors, promote tissue granulation and reepithelization, and be easy to remove [1,2]. In addition to these attributes, researchers are currently searching for dressing covers that interact with the wound and release natural bioactive molecules that provide elements necessary for wound healing [3].

Biopolymers materials, for example, are commonly used in the medical field as dressings, wound treatment, drug delivery, tissue engineering, and medical implants [4,5]. Chitosan is a natural cationic biopolymer [6] and has been considered in various biomedical applications, including wound healing, because of its many biological and physical-chemical attributes suitable for application in dressings [7,8]. Chitosan’s numerous essential advantages are related to its interaction with the tissue, causing no or low toxicity to degradation, as well as having anti-inflammatory and antibacterial effects [9,10,11]. In addition, chitosan acts actively as a hemostatic agent and supports tissue regeneration, and is therefore beneficial in wound healing [12,13].

Chitosan is composed of units of poly-(beta-1-4) N-acetyl-d-glucosamine, extracted through partial deacetylation of chitin, one of the most abundant renewable biopolymers, which is obtained at low cost and on a large scale through marine sources [14]. The chemical modifications of chitosan are associated with a tendency for changes caused by the hydroxyl and amine functional groups present in its structure [15]. However, the efficacy of its properties depends strictly on molecular weight, degree of deacetylation, polydispersibility, and its structure [16]. Therefore, chitosan can be easily processed into a variety of forms to treat skin lesions. Some of these forms, such as membranes, gels and hydrogels, nanoparticles, and scaffolds, can be effective in drug delivery with controlled release of active elements with antimicrobial actions, avoiding infections and accelerating healing [17].

Several chitosan membranes are formulated in combination with natural and synthetic base materials for wound treatment. Tamer et al. [18] formulated a chitosan-based membrane with sodium hyaluronate and used glutathione with antioxidant action to accelerate wound healing. In studies by Kenawy et al. [19], the chitosan/gelatin/cinnamaldehyde combined dressing produced membranes with effective antibacterial properties that could be used on wounds. Xie et al. [20] created a membrane composed of chitosan, collagen, and alginate with promising effects on tissue repair. A study conducted by Susanto et al. [21] concluded that the chitosan membrane associated with collagen induced an increase in the number of fibroblasts in the mandibular defect of rats.

Medicinal alternative therapies for treating wounds with bioactive compounds have gained importance in recent years [22]. Combining chitosan with copaiba oil can be an attractive compound to minimize wound healing difficulties, and prevent infections.

Copaiba oleoresin is an abundant natural resource in the Brazilian Amazon, used for centuries by traditional peoples for therapeutic purposes, mainly anti-inflammatory and wound healing [23,24,25]. The medicinal property of copaiba oil was attributed to β-caryophyllene, one of the most important constituents of the oil. Several studies have reported β-caryophyllene as possibly responsible for the anti-inflammatory action [26,27]. Moreover, other pharmacological properties have been attributed to the oil, such as analgesic, antinociceptive, antitumor, antioxidant, and antimicrobial activities [28,29,30]. The antibacterial effect was potentially observed against the pathogen *Staphylococcus* *aureus* by combining chitosan/gelatin/copaiba oil in the form of an emulsion [31].

Biopolymers’ properties can be enhanced or improved with the incorporation of copaiba oil. Norcino et al. [32] concluded that pectin films loaded with copaíba oil nanoemulsions improved physical-mechanical and antimicrobial properties. Experiments by Pascoal et al. [33] resulted in developing topical dressings with copaiba oil conjugated with commercial biopolymers as an option for the controlled release of cutaneous anti-leishmaniasis drugs. Hydrogel formulation based on polymers containing copaiba oil nanoemulsions reduced the inflammatory factors caused by ear and paw edema in mice [34]. Nanocapsules containing copaiba oil and chitosan may increase synergism with anti-cancer drugs [35]. Chitosan-based membranes have been widely explored, especially as wound dressings [36,37,38,39,40,41]. However, there are gaps involving wound healing and appropriate materials according to the type of wounds. Studies exploring polymer synergism with copaiba oil are necessary due to incipience in the literature. Recently, Debone et al. [41] researched different concentrations of chitosan-copaiba oil films for wound dressing application, but in this work, the authors did not investigate the combination of various copaiba oil concentrations (0.1, 0.5, 1.0, and 5.0% *v*/*v*) in chitosan solution (1.0% *v*/*v*). Furthermore, [41] did not research the contact angle, moisture content, and X-ray diffraction of films. Moreover, [41] did not perform statistical analysis to validate the results. In the present work, a detailed statistical analysis of the results of contact angle and moisture content was done, and the interaction of chitosan and copaiba oil was for the first time studied in different concentrations as an approach to develop a membrane that could be beneficial as an active dressing for skin wounds. In this context, the objective of this study is to analyze the influence of copaiba oil on the chitosan membrane as a possible application in the treatment of skin wounds.

## 2. Materials and Methods

### 2.1. Materials

Commercial chitosan (CAS: 9012–76–4) (medium molecular weight-190,000–310,000 Da and deacetylation degree in the range 75–85%) was obtained from Sigma Aldrich Laboratory (São Paulo, Brazil). Glacial acetic acid (Scientific Exodus), sodium hydroxide P.A. (Vetec) reagents, and distilled water (conductivity 1.5 μS/cm). Phosphate buffered saline solution (PBS) with pH = 7.2. The copaiba resin oil was obtained from Amazon Oil (Belém, Brazil). Its technical characteristics are listed in Table 1.

### 2.2. Membrane Production by Casting Method

The emulsions for membrane production were prepared following the method described by [41] with modifications. For the production of chitosan suspension, the polysaccharide (1.0% *v*/*v*) was dissolved in an aqueous solution of acetic acid (1% *v*/*v*). To verify the effective formation of the emulsion, solutions were kept at rest for 7 days, and then a visual analysis was carried out in order to verify the non-separation of the phases.

**Figure 1:**
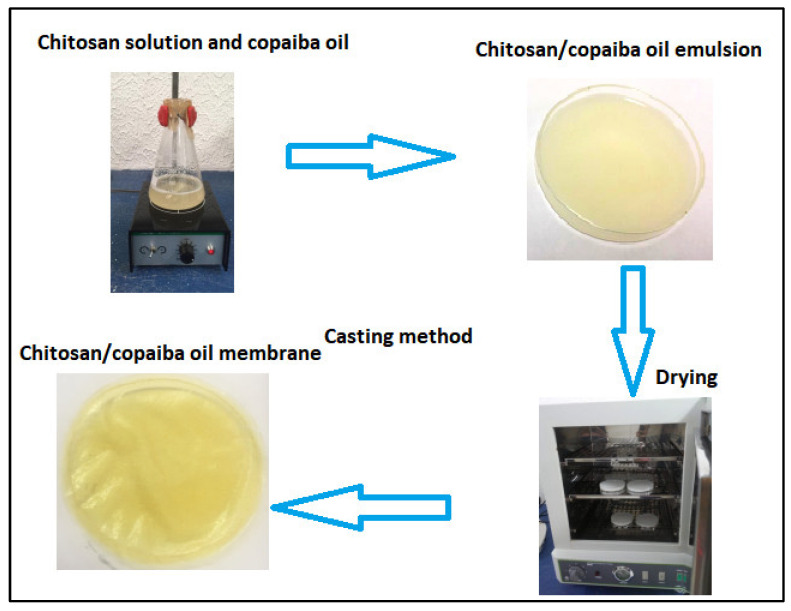
Schematic diagram of the synthesis of chitosan membranes/copaiba oil by the casting method.

The membranes were obtained by the casting method (see Figure 1) according to the methodology proposed by [41] with modification. Chitosan was dissolved in an aqueous solution of acetic acid in magnetic agitation for 24 h. Copaiba oil was added to the chitosan solution in magnetic agitation for another 3 h. Four concentrations of copaiba oil (0.1%, 0.5%, 1.0%, and 5% *v*/*v*) were analyzed in chitosan solution. After stirring, the mixture (chitosan/oil solution) was spilled into a polystyrene Petri dish with a volume of 50 mL. The solutions were subjected to a drying process for 24 h on a stove at 40 °C for both acid evaporation and membrane formation. Dry membranes were neutralized with 5% sodium hydroxide (*v*/*v*) for 2 h. After this period, the membranes were washed with plenty of distilled water to remove the excess from the reagent and then placed for drying at room temperature (RT) for 24 h. The chitosan membrane without copaiba oil was produced as a control sample and subjected to the same processing conditions.

### 2.3. Characterization of Membranes

#### 2.3.1. Macroscopic Analysis

The chitosan membranes prepared with and without copaiba oil were subjected to visual and tactile inspection to evaluate color, continuity, homogeneity, and maneuverability.

#### 2.3.2. Scanning Electron Microscopy (SEM)

The surface of the membranes was observed using a scanning electron microscope (Tescan-Vega 3, Brno, Czech Republic) equipped with a 5 kV field emission electron source. Initially, the membranes cryogenic cut on liquid nitrogen then mounted on a metal smear and coated with a thin layer of gold–palladium. The particle diameter was measured using the Vega 3 software, model Vega 3 (Tescan, version 3, Czech Republic).

#### 2.3.3. Absorption Capacity/Degree of Intumescence

Tests for the absorption capacity or degree of intumescence of Ch-control membranes ChCo-0.1, ChCo-0.5, ChCo-1.0, ChCo-5.0 samples with a 1 cm diameter were performed in phosphate-saline buffer solution (pH = 7.4) and in distilled water at RT in triplicate to evaluate the initial behavior of intumescence in the first hour and after 24 h, 7 days, and 14 days, which corresponds to the time of the future biocompatibility study. The samples were initially weighed on a digital analytical scale (ChyoBrand, jk 200 model, Tokio, Japan) and then immersed in fluids. After each period of analysis, the tumid membranes were removed from the medium, and the excess liquid was removed from the surface using filter paper. The absorption capacity was determined by means of the difference of the tumid samples in relation to the dried samples according to:

Absorption (%) = ((Mf − Mi)/Mi) × 100(1)

where: Mf = final mass of the tumid sample; Mi = dry initial sample mass.

#### 2.3.4. Moisture Determination

The moisture content of the membranes was evaluated by the gravimetric method. For this, the initial mass of the samples of the three membranes was weighed on the above-mentioned analytical scale. Then, the samples were dried in an analogical stove (Sterilifer-modelSX1.2, São Paulo, Brazil) at ±105 °C for 24 h. After this procedure, they were again weighed. The humidity contained in the samples was determined according to:

U (%) = ((Mi − Mf)/Mi) × 100(2)

where: U = percentage of humidity; Mi = initial mass; Mf = final mass.

#### 2.3.5. Contact Angle Measurement

The contact angle of the droplet with the membrane was determined on each sample using the sessile drop method. A drop (0.05 mL) of distilled water was placed on the membrane at RT for 30 s. Images were captured with a camera and analyzed by the ImageJ software (National Institute Health, version 1.51n) to measure the angle formed between the membrane contact and water drop.

#### 2.3.6. X-ray Diffractometry (XRD) Analysis

XRD analyses were performed on the Empyrean Model X-Ray diffractometer (DXR) (Malvern, United Kingdom) of PANalytical using Co anode ceramic X-ray tubes (Kα1 = 1.789010 Å). Phase identification was performed using the X-Pert HighScore Plus (Panalytical) software (Malvern Panalytical, version 5.1).

#### 2.3.7. Statistical Analysis

The statistical validation of the data was performed using the analysis of variance test (ANOVA), with a confidence interval of 95% (*p* < 0.05). The mean values were compared by the Tukey test.

## 3. Results and Discussion

### 3.1. Macroscopic Analysis of Membranes

Table 2 shows the macroscopic aspects of the membranes.

All chitosan solutions produced thin, yellowish membranes. The pure chitosan membrane (without oil) exhibited a slightly yellow color and relative transparency. The yellow color was more intense in the membranes prepared with copaiba oil. Such aspects of the color of the membranes are related to both the color of chitosan and the direct influence of the natural color of the oil. Thus, the yellowish color of the membranes is directly proportional to the concentration of copaiba oil in the chitosan matrix. Moreover, the chitosan membranes with higher copaiba oil contents not only modified the color of the polymer but left them less transparent. However, at lower levels of copaiba oil, the color and transparency seemed similar. Similar effects were observed for chitosan films containing two concentrations of copaiba oil (1% and 2% *v*/*v*), in which a significant color difference for films more than 2% chitosan were formed for higher oil concentration [41]. The electrostatic interaction between the protonated amino groups of chitosan and the molecules of copaiba oil might have induced this significant change in color with the increase in oil concentration [42].

Based on these results, it can be confirmed that Ch-control and membranes prepared with 0.1%, 0.5% and 1% copaiba oil presented better macroscopic characteristics such as good solubility, no fractures or ruptures, and easy handling. However, the chitosan membrane with 5% copaiba oil (ChCo-5.0) seems to be less appropriate, as it presented fragility during the detachment of the petri dish and caused the separation of phases. This separation seen macroscopically on the membrane surface suggests that the polymer did not completely incorporate the oil into its structure, apparently because it is the membrane with the highest amount of copaiba oil.

### 3.2. SEM

**Figure 2:**
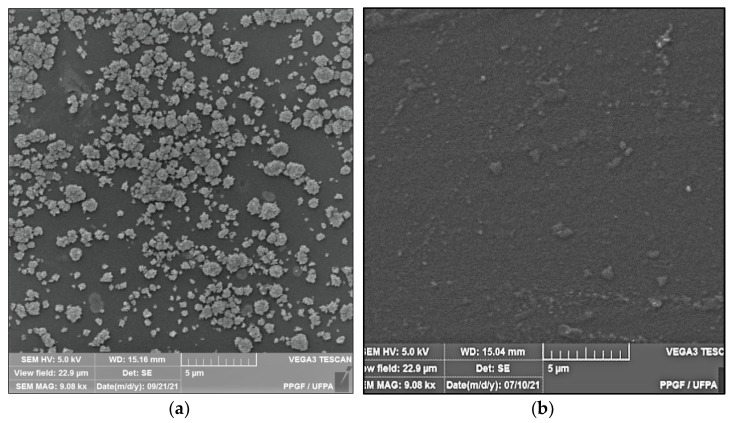

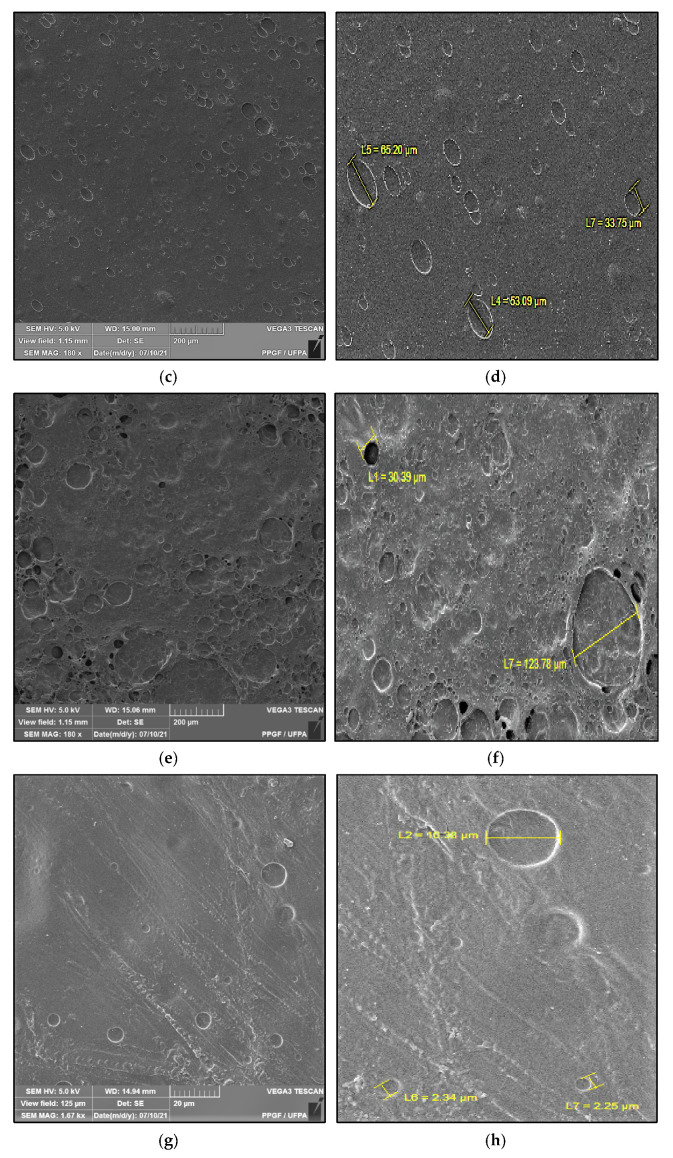

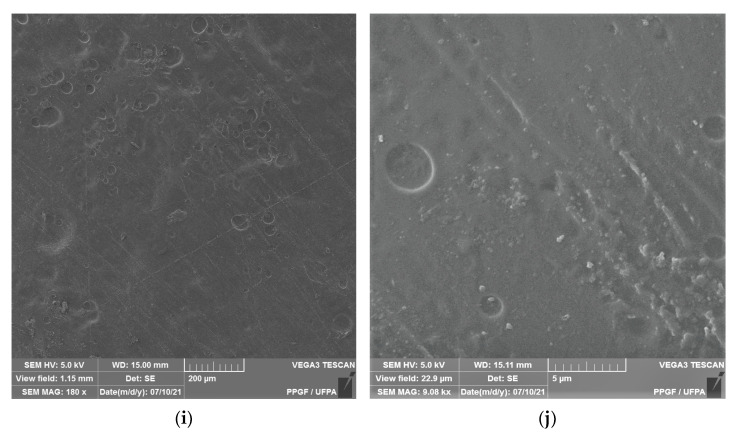
Membrane micrographs: (**a**) oil-free chitosan (Ch-control)180 magnification; (**b**) Oil-free chitosan (Ch-control) magnification of 9.08; (**c**) chitosan with 0.1% copaiba oil (ChCo-0.1) 180 magnification; (**d**) chitosan with 0.1% copaiba oil (ChCo-0.1) magnification of 9.08; (**e**) chitosan with 0.5% copaiba oil (ChCo-0.5) magnification of 180, (**f**) chitosan with 0.5% copaiba oil (ChCo-0.5) magnification of 9.08; (**g**) chitosan with 1.0% copaiba oil (ChCo-1)magnification of 180; (**h**) chitosan with 1.0% copaiba oil (ChCo-1)magnification of 180; (**i**) chitosan with 5.0% copaiba oil (ChCo-5) magnification of 180; (**j**) chitosan with 5.0% copaiba oil (ChCo-5) magnification of 9.08.

Figure 2 shows the microstructure of the surfaces of Ch-control and chitosan membranes with copaiba oil (ChCo-0.1; ChCo-0.5; ChCo-1, and ChCo-5.0), and Table 3 describes the size of pore diameters.

In Figure 2c–j, the membrane matrices with copaiba oil were irregular, discontinuous, and with oil droplets, unlike the control matrix (Ch-control) that presented a rough surface. The membrane matrix (ChCo-0.1) shows a structure without the formation of appreciable oil droplets with a dense surface. On the other hand, in the membranes with 0.5%, 1%, and 5% of copaiba oil, dispersed oil drops were observed in their microstructures. However, the number of droplets was more evident in the membrane matrix (ChCo-0.5), whose droplet diameter ranged from 65.20 μm to 33.75 μm. Nevertheless, when the oil concentration was increased to 1% (ChCo-1.0), the size of the drops gained greater effects on the chitosan matrix, with diameters between 123.78 μm and 30.39 μm. In addition, it produced a cluster of drops in the chitosan matrix, promoting interconnection between the drops. This behavior can be explained due to the presence of hydroxyl groups and primary amines that provide the molecule a polar character, so there is repulsion to copaiba oil molecules and, consequently, less miscibility [43,44]. Conversely, the matrix (ChCo-5.0) revealed a structure with a smaller droplet size, which ranged from 10.38 μm to 2.25 μm. Although more pronounced, irregular characteristics were observed, which may indicate fragmentation in the microstructure.

Silva et al. [45] observed that the addition of andiroba oil decreases the number and size of pores, as the concentration of oil in poly-based polymeric film (ε-caprolactone) (PCL), as well as no oil retention on the surface of the films, which may indicate a good interaction of the compound PCL/andiroba. However, the changes that occurred in the structure of chitosan with the incorporation of oils depending on the molecular structure of the polymer and its chemical interactions. Interaction with natural oils is more favorable if there is an oil emulsification system. The might be achieved using surfactants to improve the permeability, bioavailability, and release characteristics of the active components of the oil [46]. Therefore, it is believed that there was a greater repulsion of the molecules of copaiba oil with chitosan due to the hydrophobic nature of the oil.

### 3.3. Absorption Capacity/Degree of Intumescences

**Figure 3:**
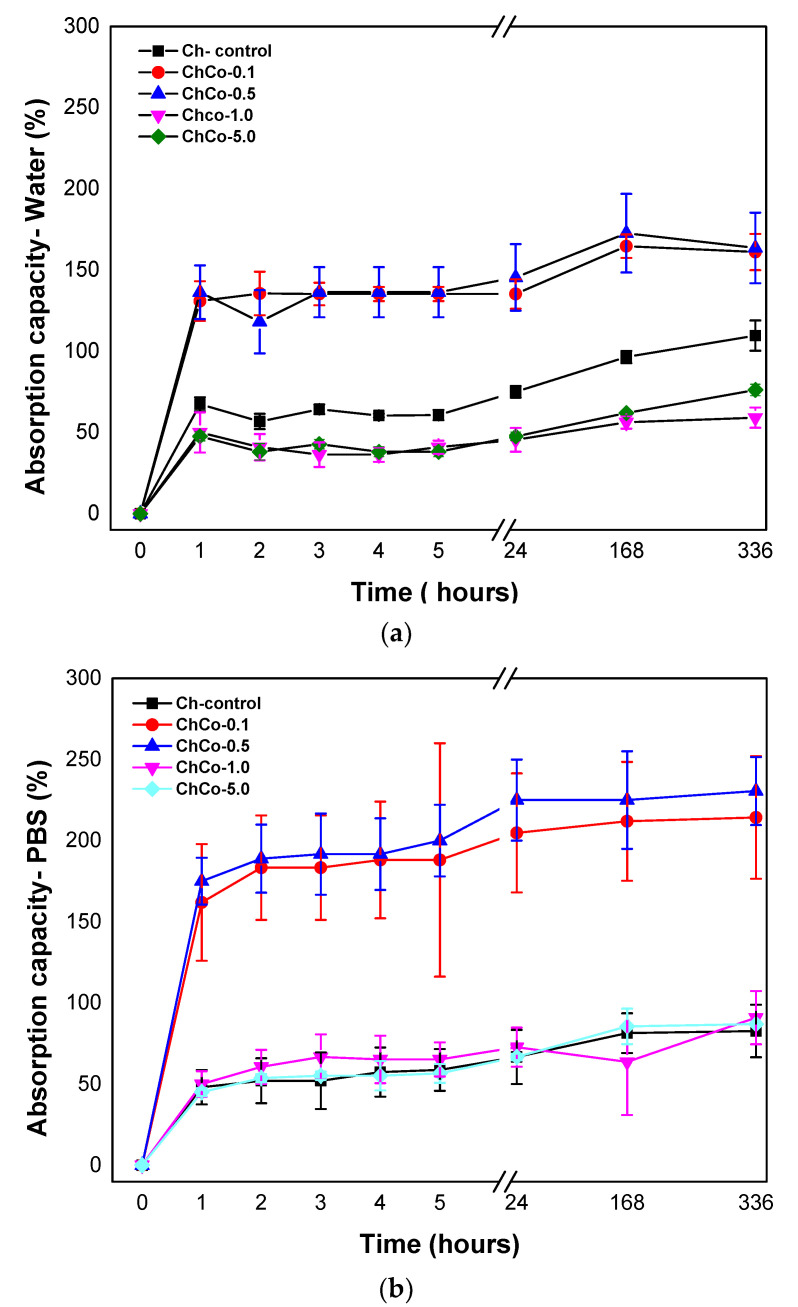
(**a**) Degree of absorption of membranes in aqueous medium as a function of time; (**b**) Degree of absorption of membranes in phosphate-saline buffer solution (PBS) as a function of time.

Figure 3 shows the degree of intumescences as a function of time for chitosan membranes without copaiba oil, and chitosan membranes with copaiba oil, after immersion in water and phosphate-saline buffer solution (PBS).

Absorbent materials are ideal for wound healing as they provide controlled moisture, avoiding infection and maceration in the wound bed. The present results showed that the membranes were able to promote fluid absorption. The high absorption rate of membranes containing copaiba oil at concentrations of 0.1% and 0.5% was evident (ChCo-0.1; ChCo-0.5). They revealed higher absorption rates in the presence of PBS solution, whose values obtained in 14 days were 214% and 230%, while in water, they reached 161% and 163%, respectively. However, high concentrations of copaiba oil in the polymer matrix (ChCo-1.0 and ChCo-5.0) showed low absorption rates, especially when in contact with water with values of 56% and 61%, and PBS solution, 90%, and 86%, respectively. However, the oil-free chitosan membrane (Ch-control) interacted better with water, obtaining enhanced absorption capacity in this medium, with a value of 109% and 82% in PBS solution in the period of 14 days. The reason for this divergence of absorption between the solutions stems from the polarity difference between water and the buffer solution. Chitosan has a strong affinity for polar molecules due to its amine and hydroxyl groups, so it has better interaction with water because it is extremely polar (pH = 5.6) when compared to the buffer solution (pH = 7.2) [42]. However, copaiba oil seems to influence the absorption behavior of the chitosan membrane when immersed in water by reducing absorption in this medium. The data also suggest that the effect of porosity may have favored the absorption capacity of membranes with low concentrations of copaiba oil in the chitosan matrix. However, as the oil concentration increases, this phenomenon is reversed due to some kind of interaction or repulsion to the oil.

Gunes and Tihminhoglu [47] concluded in their studies that the increased intumescence in the film containing *H. perfuratum* oil could be explained by the formation of a porous structure with the addition of oil in the chitosan matrix. This finding corroborates the results of the present study, as evidenced by the microstructural data obtained by SEM.

The analysis of variance, ANOVA, with *p* < 0.05 shown in Table 4, indicates a statistically significant difference in permeability between the groups of chitosan treated without copaiba oil and with copaiba oil when immersed in water and solution in PBS.

**Figure 4:**
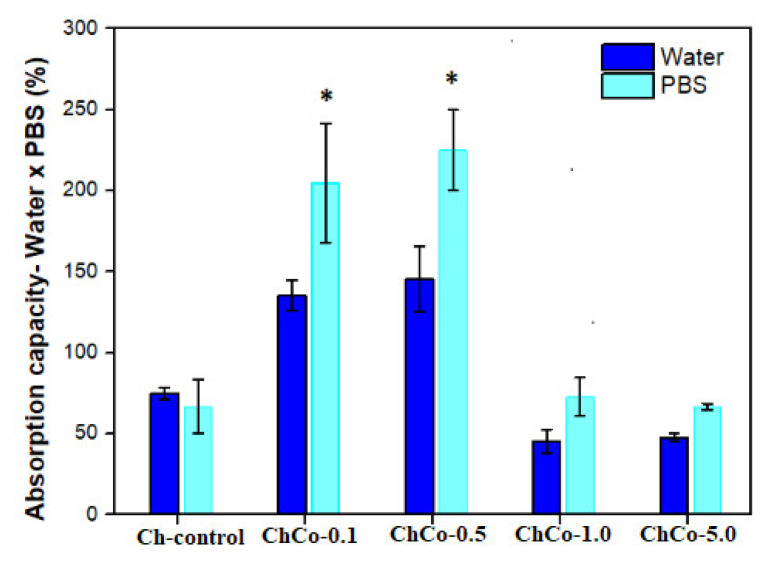
Evaluation of the absorption capacity of fluids of polymeric membranes of Ch-control without chitosan, and incorporated with copaiba oil (ChCo-0.1; ChCo-0.5, ChCo-1.0, ChCo-5.0) immersed for 24 h in water and PBS. The asterisk indicates the statistical difference between the control group and the group with oil obtained in the Tukey test.

In Figure 4, the Tukey test suggests a statistically significant difference between chitosan membranes prepared with 0.1% and 0.5% (*v*/*v*) of copaiba oil, in which greater absorption capacity was observed in the solution in PBS in relation to water. This result is favorable for materials in contact with wounds, considering that the PBS solution simulates the composition of human body fluids. There was no statistically significant difference between oil-free membranes and membranes with 1.0% and 5.0% (*v*/*v*) of copaiba oil.

In the case of dressing materials, the results may indicate that the membranes compositions ChCo-1.0 and ChCo-5.0 might be more favorable in wounds with little exudates. For example, in cases of dry wounds, this material could be used previously hydrated to dampen the site and stimulate the closure of the lesion. On the other hand, the ChCo-0.1 and ChCo-0.5 membranes may be suitable for highly exudative wounds to control local moisture and thus avoid worsening the injury due to macerated tissue caused by exudates accumulation, thus requiring frequent removal of this material in wounds.

According to the study by Bras et al. [8], a decrease in the volumetric capacity of intumescences with the incorporation of *Cynara cardunculus* extract was observed. This was attributed to the hydrophobic character of the extract and by the presence of lipid fraction in the composition, which may be appropriate for dressings where excessive sorption of fluids is not desired.

### 3.4. Contact Angle and Moisture Content Determination

Table 5 shows the measurements of the contact angle with water and moisture content for oil-free chitosan membranes and membranes with copaiba oil, aiming to investigate the surface permeability of membranes.

The wettability of the surface of a biomaterial significantly influences the interaction with the tissue that comes into contact, inducing favorable or unfavorable reactions to the organism [48]. Table 5 shows that all developed membranes presented hydrophilic surfaces, although the surface interaction with water occurred in different ways. This result can be explained by the presence of hydroxyl and amino groups present in the structure of chitosan. The positive charges that arise when the amino groups are protonated decrease the surface free energy, improving the wettability of the membranes [32]. Thus, it is assumed that copaiba oil with low contents (ChC0-0.1 and ChCo-0.5) modified the hydrophilic characteristic of membranes, reducing surface hydrophilicity. However, 1.0% oil content enhanced the membrane hydrophilicity. Such behavior can be associated with the equilibrium relationship between the adhesive and cohesive forces of the liquid, suggesting that a change in this balance may have caused a change in the droplet diameter and, consequently, an increase in hydrophilicity as indicated by [15]. Meanwhile, the hydrophilic condition of the sample with 5% oil (ChCo-5%) approached that of the control membrane. Similar results [32] occurred in the formation of the film containing copaiba oil and pectin, which caused an increase in the contact angle and consequently resulted in a decrease in the hydrophilicity of the polymeric film.

Table 5 presents values of the moisture content of the membranes. The developed membranes disclose different percentages of moisture. However, the oilless chitosan membrane (Ch-control) is shown to be relatively moister between the membranes. This behavior decreased in membranes containing copaiba oil, making them less moist.

ANOVA showed a hydrophilicity effect associated with the surface of chitosan membrane with copaiba oil since it showed a statistically significant difference between oil-free membranes and oil-treated membranes, as shown in Table 6.

**Figure 5:**
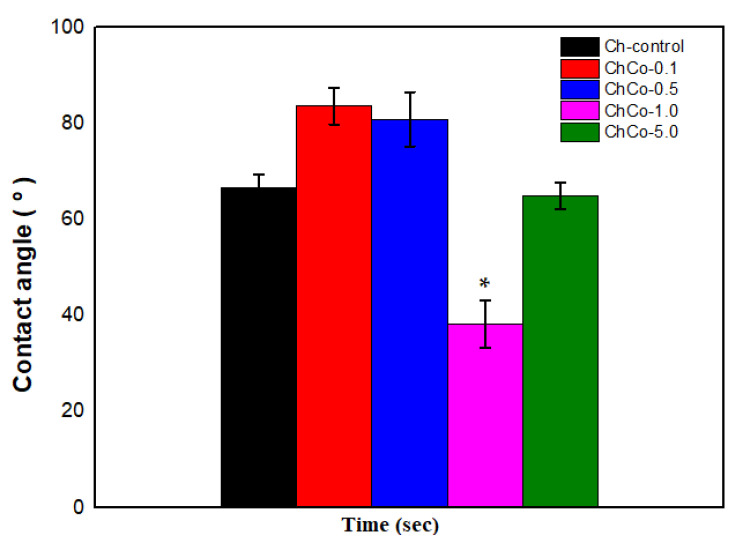
Contact angle of membranes with analysis of the Anova Test (*p* < 0.05; DF = 4) and the Tukey Test. The asterisk indicates the sample with significant statistical difference (*p* < 0.05).

In Figure 5, the Tukey Test confirms that the chitosan membrane with 1.0% copaiba oil was the most hydrophilic sample with the lowest contact angle with a significant statistical difference (*p* < 0.05) between the membranes. In contrast, samples with low copaiba oil content have a less hydrophilic character when compared to the control sample and the sample with 5% (*v*/*v*) of oil.

As was observed by the ANOVA, there is a statistical difference between membranes. Indeed, there is an effect on the membrane moisture when copaiba oil is incorporated in the polymer matrix of chitosan, as shown in Table 7.

**Figure 6:**
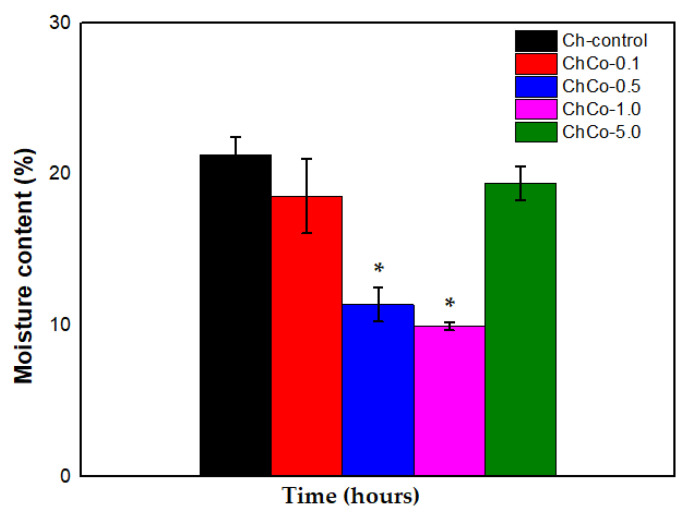
Percentage of membrane moisture with analysis of the ANOVA Test (*p* < 0.05; DF = 4) and the Tukey Test. The asterisk indicates the sample with significant statistical difference (*p* < 0.05).

In Figure 6, the Tukey Test showed that chitosan membranes containing 0.5% and 1.0% showed a statistically significant difference between membranes (*p* < 0.05). Thus, the data suggest that the water content was lower in the ChCo-0.5 and ChCo-1.0 membranes. However, chitosan membranes with 0.1% and 5.0% copaiba oil lost little water in their structures compared to the control chitosan membrane (Ch-control).

### 3.5. X-ray Diffractometry (XRD) Analysis

**Figure 7:**
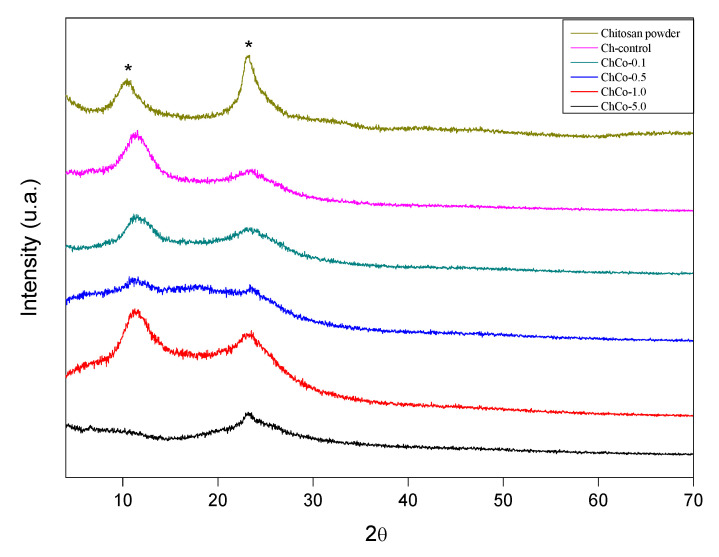
X-ray diffractogram. (*) Chitosan.

The diffractogram of the studied membranes is shown in Figure 7. This figure shows that the addition of copaiba oil causes a broadening of the peaks of chitosan without oil, whose diffractogram is characteristic of a semi-crystalline material.

The diffractions of 2 broad peaks in 2θ: 10.55, 23.373, 2θ: 11.55, and 23.70, respectively, were analyzed. Such peaks are typically chitosan. Chitosan arrangements with copaiba oil promote enlargement of the peaks of the polymer matrix, resulting in a decreased crystallinity. In the chitosan sample with 0.1% copaiba oil, only 1 peak was observed at 2θ: 22.96. In samples containing 0.5% and 1.0% copaiba oil, diffraction peaks were not identified, characterizing them as an amorphous structure. There was a discrete peak in 2θ in the sample containing 5.0% copaiba oil in 2θ: 7.92. Similar behavior was observed in the interaction between cyclodextrin and copaiba oil, causing changes in the XRD profile resulting in the formation of inclusion complexes [49]. Tovar et al. [50] observed that the crystalline structure of chitosan was slightly affected by the incorporation of *Ruta graveolens* essential oil.

## 4. Conclusions

Polymeric membranes composed of chitosan and copaiba oil were successfully developed by the casting method for application in wound treatment. The incorporation of copaiba oil in the polymer matrix produced membranes. These membranes can be applied to wounds with different exudative characteristics, such as ChCo0.1 and ChCo0.5% indicated for more exudative wounds, and ChCo1.0 and ChCo5.0% for less exudative wounds. The membrane structure presented interfacial matrices with favorable porosity and wettability, which may have improved fluid absorption, strongly dependent on oil concentrations. In addition, copaiba oil produced an interaction with chitosan, altering its crystallinity pattern. These results indicate that the influence of copaiba oil on the chitosan matrix might be favorable in wound healing. However, further studies of the mechanical properties and tests of antimicrobial and cytotoxic activities are necessary to ensure the safety and use efficacy as a biomaterial.

## Figures and Tables

**Table 1:** Technical specifications of commercially purchased copaiba oil.

Characteristics	Units	Presentation
Appearance (25 °C)	--------	Liquid, with low viscosity
Color	--------	Yellowish, greenish to brown
Aroma	--------	Typical wooden
Density	g/mL	0.912
Refraction index (20 °C)	--------	1.5
Solubility in water	--------	Insoluble

**Table 2:** Macroscopic characteristics of membranes.

Description		Color	Homogeneity	Continuity	Handling
Ch-control	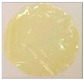	Slightly yellowish	Loud	Loud	Loud
ChCo-0.1	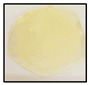	Slightly yellowish	Intermediate	Loud	Loud
ChCo-0.5	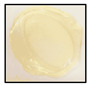	Slightly yellowish	Intermediate	Loud	Loud
ChCo-1.0	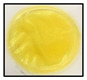	More intense yellow+	Intermediate	Loud	Loud
ChCo-5.0	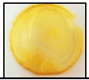	More intense yellow++	Low	Low	Low

**Table 3:** Size of membrane pores.

Description	>Diameter (μm)	<Diameter (μm)
Ch-control	-	-
ChCo-0.1	-	-
ChCo-0.5	65.20	33.75
ChCo-1.0	123.78	30.39
ChCo-5.0	10.38	2.25

**Table 4:** Results of variance analysis (ANOVA), performed to evaluate the absorption capacity of membranes with copaiba oil content on the chitosan matrix in water and PBS solution.

Origin of Variation	Sum of Squares	Degrees of Freedom (DF)	Middle Square	Value of F	*p*-Value
Between groups	493,634	9	54,848.20	30.2	<0.05
Within two groups	364,143	200	1820.71	-	-
Total	857,777	209	0.00001		

**Table 5:** Measurements of the contact angle with water and moisture content of the developed membranes.

Membranes		Contact Angle (°)	Moisture Content (%)
Control chitosan membrane	Ch-control	66.73 ± 2.56	21.28 ± 1.18
Chitosan membrane with 0.1% copaiba oil	ChCo-0.1	83.63 ± 3.78	18.57 ± 2.45
Chitosan membrane with 0.5% copaiba oil	ChCo-0.5	80.90 ± 5.65	11.39 ± 1.13
Chitosan membrane with 1% copaiba oil	ChCo-1.0	38.33 ± 4.97	9.93 ± 0.25
Chitosan membrane with 5% copaiba oil	ChCo-5.0	65.03 ± 2.77	19.42 ± 1.12

**Table 6:** Results of variance analysis (ANOVA) performed to evaluate the effect of hydrophilicity on the surface of membranes with copaiba oil content on the chitosan matrix.

Origin of Variation	Sum of Squares	Degrees of Freedom (DF)	Middle Square	Value of F	*p*-Value
Between groups	3886.70	4	971.68	56.93	<0.05
Within two groups	170.69	10	17.07	-	-
Total	4057.39	14	0.00008		

**Table 7:** Results of variance analysis (ANOVA) performed to evaluate the effect of moisture of membranes with copaiba oil content on the chitosan matrix.

Origin of Variation	Sum of Squares	Degrees of Freedom (DF)	Middle Square	Value of F	*p*-Value
Between groups	312.31	4	78.0769	38.78	<0.05
Within two groups	20.13	10	2.01318	-	-
Total	332.44	14	0.00027		

## Data Availability

Not applicable.
